# Low-grade dietary-related inflammation and survival after colorectal cancer surgery

**DOI:** 10.1007/s00432-014-1711-6

**Published:** 2014-05-27

**Authors:** Aleksander Galas, Jan Kulig

**Affiliations:** 1 Department of Epidemiology, Chair of Epidemiology and Preventive Medicine, Jagiellonian University Medical College, 7a Kopernika St, 31-034 Kraków, Poland; 2 I Chair of General Surgery and Department of Gastroenterological Surgery, Jagiellonian University Medical College, 40 Kopernika St, 31-501 Kraków, Poland

**Keywords:** Diet, Anti-inflammatory agents, Colorectal cancer, Survival, Determinant

## Abstract

**Purpose:**

Prolong inflammation is a central process observed in several chronic conditions and may be responsible for survival. There is an increasing evidence showing the role of diet in inflammation and habitual diet may be responsible for low-grade inflammation. The purpose of our study was to assess the effect of inflammatory properties of habitual diet measured by the Dietary Inflammatory Index (DII) on survival among surgical patients treated for colorectal cancer (CRC).

**Methods:**

A follow-up study among 689 CRC patients (mean age 58 years, ±8.9; 56.7 % males) treated surgically was performed in Krakow, Poland. Habitual diet was assessed by a standardized semiquantitative food frequency questionnaire. Next, 23 dietary items were used to calculate DIIs. Vital records were verified to determine status of the participants.

**Results:**

Study has shown linear association between DII and survival time among CRC patients with totally removed cancer treated by chemotherapy (*b* = −0.13, *p* = 0.024). After adjustment for several important covariates, DII was associated with survival during up to 3 years after surgery, but only in patients without distant metastases (3-year HR_DII>−2.27_ = 0.61, 95 % CI 0.38–0.99).

**Conclusions:**

The results of the investigation have shown the usefulness of the DII as a potential predictor of survival among patients without distant metastases treated surgically for CRC.

## Introduction

Inflammation has been recognized as an important property of several chronic conditions. It has been observed that several inflammatory biomarkers, that is, IL-1β, IL-4, IL-6, IL-10, TNF-α and C-reactive protein (CRP) are potential markers of increased risk of cardiovascular endpoints (Koenig 2013; Halcox et al. 2014), metabolic syndrome (Fuentes et al. 2013), lung (Guo et al. 2013) and colorectal cancer (CRC) (Wu et al. 2013; Song et al. 2013), and some other chronic diseases (Iyengar et al. 2013; Rodriguez-Hernandez et al. 2013).

Diet is one of a key element of individual’s lifestyle responsible for the development of chronic diseases. For the last few years, an increased interest in diet as a determinant related to, and even causally associated with, the level of inflammation has been observed. Dietary patterns have been linked with some biomarkers of low-grade inflammation (Barbaresko et al. 2013). Western type diet was associated with an increase in CRP, IL-6 and serum amyloid A, whereas ‘healthy type’ diet (high in fruits, vegetables, tomato, poultry, legumes, tea, fruit juices and whole grains), oppositely, was related with a decrease in inflammatory markers (Esmaillzadeh et al. 2007). Mediterranean diet was also assessed (Estruch 2010), and there are publications showing a decrease in inflammatory cytokines related to the increase in intake of polyunsaturated fatty acids (Calviello et al. 2013), vitamins (van Herpen-Broekmans et al. 2004) and fruits and vegetables (Root et al. 2012).

Dietary components such as macronutrients, vitamins, minerals, and trace elements may modulate the course of body inflammation and thus may change a response to injury and infection. This effect was investigated for the last years especially among malnourished patients, and it was found that diet rich in n-3 fatty acids, arginine, glutamine and vitamins C and E improves clinical outcomes and decreases the frequency of infections and infectious complications among surgical patients (Klek et al. 2011; Marano et al. 2013). This effect, however, is discussible among well-nourished patients, and results are not consistent (Barker et al. 2013; Falewee et al. 2013).

Clinical dietary intervention may improve the effectiveness of treatment; therefore, it is also reasonable to consider habitual diet as one of determinants of health outcomes. The assessment of habitual diet is very challenging. Diet is a very complex phenomenon. It includes beneficial and unfavorable constituents, and their interrelation may be responsible for the final effect. One of the solutions is a proposal to create a scoring system to assess general properties of diet. There are several dietary scores published to date; however, they have not been validated in terms either to assess inflammatory properties or to predict some inflammatory-related health outcomes. The solution may be the Dietary Inflammatory Index (DII) (Cavicchia et al. 2009). The DII is the first literature-based index to focus primarily on the inflammatory properties of diet. The Index considers pro- and anti-inflammatory dietary constituents and creates the net value, so it benefits by the assessment of general properties of diet, and thus, it seems to preponderate other scores as the Alternate Healthy Eating Index or the Alternate Mediterranean Diet Index. The DII is not dependent on the population means or on the recommendations of intake, and it is not limited only to macronutrients and micronutrients, but also considers commonly consumed components of diet including spices, tea and others (Cavicchia et al. 2009).

The inflammation is a central process in majority diseases, and available evidence has shown that dietary habits are related to the level of inflammatory cytokines; therefore, we tried to investigate a long-term effect of dietary habits among CRC patients.

## Purpose

The purpose of the study was to assess the effect of inflammatory properties of habitual diet (understood as prolong exposure to low-grade inflammation) measured by the DII on survival among surgical patients treated for CRC.

## Materials and methods

### Study design and sample

A follow-up prospective cohort study included CRC patients admitted to the I Chair of General Surgery and Department of Gastroenterological Surgery, Jagiellonian University Medical College, Krakow, Poland. The study was conducted between 2010 and 2013. Individuals eligible for the research were cases recruited primarily for a case-control investigation performed between 2000 and 2008 (projects 6 P05D 00220 and 2 P05D 05329) and between 2010 and 2013 (project No. N404 034039). The study design has been described elsewhere (Galas et al. 2013; Jedrychowski et al. 2009). In brief, participants were newly diagnosed incident cases of sporadic adenocarcinoma of either colon or rectum. Inclusion criteria were age of up to 75 years, Caucasian, being a native Polish, referred to a surgical treatment of CRC. Exclusion criteria were the following: a presence of communication (verbal contact) problems and/or cognitive limitations or a diagnosis of any of the following: hereditary non-polyposis CRC, familiar adenomatous polyposis, attenuated familial adenomatous polyposis, mixed polyposis syndrome, Ashkenazi colon cancer, hereditary breast and CRC or any of hamartomatous polyposis syndromes, moreover, a diagnosis of secondary cancer (its distant metastasis in large bowel), a diagnosis of primary cancer other than colorectal, a recurrent cancer or underwent surgery (before recruitment) of gastrointestinal tract, present or past diagnosis of chronic disease of gastrointestinal tract (diverticulitis, irritable bowel syndrome, acute or chronic gastric ulcer, acute/chronic pancreatitis), diabetes (any type), renal failure, hepatic insufficiency or a presence of prolonged gastrointestinal symptoms. The diagnosis of colon either rectal cancer in all study participants was confirmed histopathologically.

In total, 703 cases were recruited for the study. Next, 14 subjects were excluded from the analyses due to lack of information about lifestyle factors (not willing to finish an interview), a refusal to undergo surgery treatment, no access to detailed information about hospitalization or being a patient admitted for reoperation. Finally, the remaining 689 cases (98.0 % of the recruited sample) were analyzed.

### Tools and data collection

Study participants were interviewed after the admission to the hospital, but before surgery about their dietary habits, some demographic characteristics and other potential covariates. Clinical information was collected from hospital medical records. Dietary habits were assessed by a semiquantitative food frequency questionnaire (SFFQ) developed in cooperation with the German Cancer Research Centre, during introductory part of the European Prospective Investigation into Cancer and Nutrition (EPIC-Potsdam) project. In total, 148 dietary items were used including questions about consumption of cereals, dairy products, bread, type and cuts of meat and fish, fresh fruits (summer/winter time), salads and fresh and cooked vegetables, rice or pasta, soups, sweets, baked goods, drinks and others. For each food or beverage item, a commonly consumed portion size was specified by standardized photographs. Next, respondents were asked to provide information about frequency of consumption. For the research, information about usual (habitual) consumption over the period of 1 year by calendar seasons was gathered by trained interviewers. Patient cases were asked about their dietary patterns at a time of 5 years prior to the onset of gastrointestinal symptoms (if present) or prior to the beginning of the diagnosis process; this was done to assess a habitual dietary pattern of participants. Dietary data were recalculated using Polish food composition tables to obtain information about an average consumption of dietary macro- and micronutrients (Kunachowicz et al. 1998; Nadolna et al. 2000). The validity and reproducibility of the questionnaire was assessed and was published elsewhere (Bohlscheid-Thomas et al. 1997a, b).

*Inflammatory properties of diet* have been assessed by the DII developed by Cavicchia and coworkers (Cavicchia et al. 2009). The DII considers different nutritional data, including caloric intake, consumption of different beverages and alcoholic drinks, intake of macro and micronutrients, including vitamins, microelements and others. Dietary data collected in the current investigation allow calculation of the following: an average intake of energy, drinking of tea, consumption of caffeine, different alcoholic drinks such as wine, beer and liquor (for the purpose of the study, wine and beer consumption was considered directly, and other sources of alcohol such as fruit wine, sparking wine and vodka were recalculated according to their content of pure alcohol, and as thus they were used in the calculation of the DII) and intake of carbohydrates, fiber, fat, n-3 and n-6 fatty acids, monounsaturated fatty acids (MUFA), saturated fatty acids, protein, cholesterol, vitamin A, thiamin, riboflavin, niacin, vitamin C, vitamin E, β-carotene and iron. All in all, it was possible to obtain the DII based on the 23 different dietary items in our study. The DII measure was obtained as a sum of adjusted scores as they were published by Cavicchia et al. (2009) (see “Appendix”), and finally, it provided the average habitual DII for every individual studied. Higher DII described better diet, meaning with a lower content of pro-inflammatory and a higher content of anti-inflammatory dietary components.

### Survival

Vital records were used to determine participants’ status, and the date of death was collected for every deceased patient. Vital status was verified three times between 2010 and 2013, and the last check took place in May 2013 and covered deaths occurring until the end of January 2013.

### Covariates

Considering that survival rate among patients treated for CRC is determined by several factors, several covariates were used for adjustment, including: (1) age in years (considered as continuous variable); (2) marital status (married or other); (3) overweight or obesity (understood as body mass index ≥25 kg/m^2^); (4) smoking status at a time of surgery (non-smoker, ex-smoker or active smoker); (5) calendar year when surgery was performed; (6) surgery type (radical or palliative); (7) cancer site (colon or rectum); (8) chemotherapy after surgery (yes/no); (9) radiotherapy after surgery (yes/no).

### Statistical analysis

As a first step of investigation, the DII for every individual was calculated as described above. Next, to provide basic characteristics of the study group, general descriptive data and differences across groups of patients who survived and those who died during the study period have been presented. In order to test differences between groups, the normality of the distribution for continuous variables was tested by Shapiro–Wilk test. As the distributions were not normal, the *U*-Mann–Whitney test was used to assess significance. Characteristics in categorical scale were compared across groups by the Chi-square test, because every expected count was higher than five. The main determinant of survival is the presence of distant metastases, thus a role of low-grade dietary-related inflammation (measured by the DII) was investigated separately among patients without and patients with metastases. In order to estimate hazard ratios (HRs) multivariable Cox’s regression models were used. HRs were assessed for 1-, 3- and 5-year observation period as well as for the whole follow-up. Because the DII is a relatively new index, it was tested as a continuous and as a categorical variable with a cutoff equal to the median observed in the group of survivors. Finally, Kaplan–Meier survival curves and Nelson-Aalen cumulative hazard functions for death have been drawn to show graphically the differences between DII groups over time.

The study was conducted in accordance with the ethical principles of the Declaration of Helsinki and was approved by the Bioethical Committee of Jagiellonian University (number KBET/115/B/2011).

The analyses were done by the Stata/IC 11.1 for Windows (64-bit × 86-64) StataCorp LP software. Results with the *p* < 0.05 were considered statistically significant.

## Results

The cohort comprised 689 patients included in the analysis for follow-up study. All observed subjects accounted for 3,180.31 person-years. Three hundred and nine (44.8 %) patients died during the study time. There were 56.7 % of men in the study group. Gender distribution across groups was almost the same among deceased and alive patients. Considering background characteristics, patients who died were at similar age to survivors, but had different distribution of smoking (higher proportion of current smokers: 27.8 vs. 22.9 %), had more frequently distant metastases (51.5 vs. 5.0 %), more frequently underwent palliative surgery (17.5 vs. 1.8 %) and in higher proportion were referred to postsurgical chemotherapy (83.8 vs. 55.3 %) (Table 1).

**Table 1:** Basic characteristics of the study group across vital status during study period

	Total [*n* = 689]	Alive [*n* = 380]	Died [*n* = 309]	*p*
Males [%, (*n*)]	56.7 % (391)	56.3 % (214)	57.3 % (177)	*df* = 1 *p* ^Chi^ = 0.935
Age (years)				
Mean (SD)	58.0 (8.9)	58.1 (8.8)	57.9 (9.1)	
Median, Q1–Q3	59 (52–65)	59 (52–65)	59 (53–65)	*p* ^MW^ = 0.910
Married				
Yes	86.5 % (596)	7.9 % (334)	84.8 % (262)	*df* = 1 *p* ^Chi^ = 0.236
No	13.5 % (93)	12.1 % (46)	15.2 % (47)	
Smoking [%, (*n*)]				
Non-smokers	44.1 % (304)	42.1 % (160)	46.6 % (144)	
Ex-smokers	30.8 % (212)	35.0 % (133)	25.6 % (79)	*df* = 2 *p* ^Chi^ = 0.025
Current smokers	25.1 % (173)	22.9 % (87)	27.8 % (86)	
Overweight or obesity (BMI ≥25 kg/m^2^) [%, (n)]	69.5 % (478)	70.2 % (266)	68.6 % (212)	*df* = 1 *p* ^Chi^ = 0.655
Dietary Inflammatory Index				
Mean (SD)	−2.66 (2.43)	−2.57 (2.48)	−2.77 (2.37)	
Median, Q1–Q3	−2.42 (−3.91 to −1.15)	−2.27 (−3.73 to −1.08)	−2.65 (−3.99 to −1.18)	*p* ^MW^ = 0.112
Cancer site [%, (n)]				
Colon	48.2 % (332)	47.9 % (182)	48.5 % (150)	*df* = 1 *p* ^Chi^ = 0.865
Rectum	51.8 % (357)	52.1 % (198)	51.5 % (159)	
Distant metastasis				
No	74.2 % (511)	95.0 % (361)	48.5 % (150)	*df* = 1 *p* ^Chi^ < 0.001
Yes	25.8 % (178)	5.0 % (19)	51.5 % (159)	
Surgery type				
Radical	91.1 % (628)	98.2 % (373)	82.5 % (255)	*df* = 1 *p* ^Chi^ < 0.001
Palliative	8.9 % (61)	1.8 % (7)	17.5 % (54)	
Chemotherapy [%, (*n*)]				
No	30.8 % (212)	43.2 % (164)	15.5 % (48)	
Yes	68.1 % (469)	55.3 % (210)	83.8 % (259)	*df* = 2 *p* ^Chi^ < 0.001
Referred to but not confirmed	1.2 % (8)	1.6 % (6)	0.7 % (2)	
Radiotherapy [%, (*n*)]				
No	97.7 % (673)	97.9 % (372)	97.4 % (301)	*df* = 1 *p* ^Chi^ = 0.675
Yes	2.3 % (16)	2.1 % (8)	2.6 % (8)	

Chi-square test, *MW* the *U*-Mann–Whitney test

**Fig. 1:**
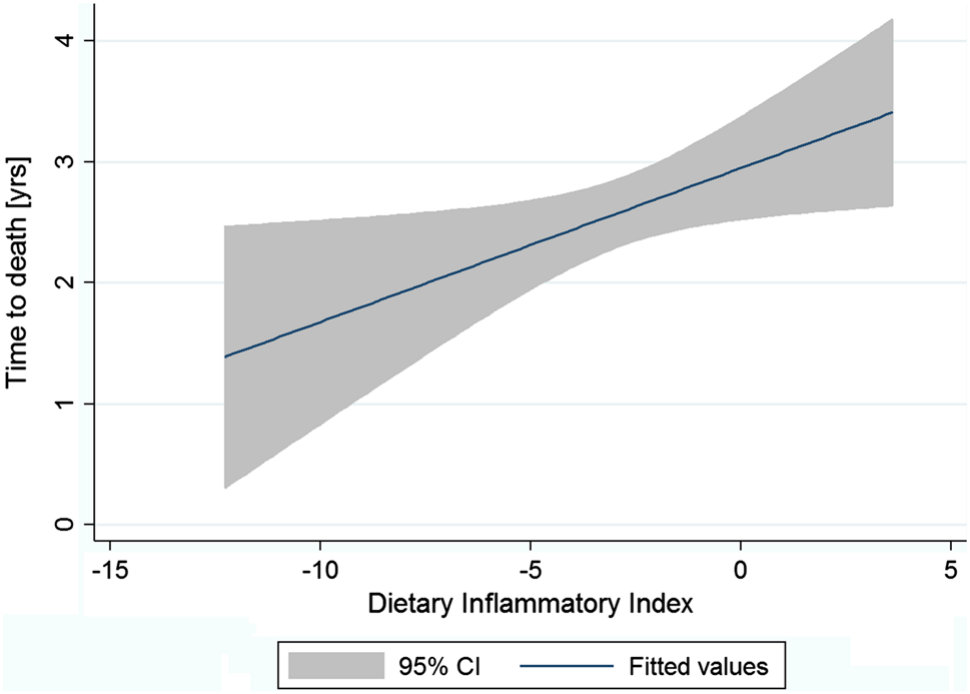
Univariable linear regression between the DII and survival time among patients who died during observation period [*n* = 215, survival time (years = 2.95 + 0.13 × DII; *p* = 0.024]. *Analysis limited to patients who underwent total surgery and were treated by chemotherapy

The DII was used to assess the role of diet as a possible determinant of survival. Overall, the mean DII was −2.66 (SD = 2.43) and median: −2.42 (IQR −3.91 to −1.15) with a slightly higher mean value observed among survivors. As the DII was expected to predict survival time after surgical treatment, we decided to use linear regression to test the association between the DII and the survival time. We performed the analysis among all patients who died during study time, and the result was not significant (*p* = 0.174). Considering that some characteristics might influence survival time, subsequently we limited a sample to the group of these who underwent total surgery and were treated with chemotherapy. Consequently, we observed a linear association between the DII and the survival time (an increase by 0.13 years in survival for every increase in the DII by 1 point; *p* = 0.024) (Fig. 1).

Finally, we used Cox regression models to assess the role of the DII as a possible determinant of survival. After adjustment for important covariates, we have found that the risk of death was decreased among patients without distant metastases and with higher DIIs during 1 year after surgery if the DII was considered either as a continuous (HR = 0.87; *p* = 0.048) or as a categorical variable (1-year HR_DII>−2.27_ = 0.40; *p* = 0.050). After 3-year time the risk was still decreased for the patients with the DII above the median (3-year HR_DII>−2.27_ = 0.61; *p* = 0.046) (Table 2). The effect of the DII was not observed among patients with distant metastases, independently from the period of time.

**Table 2:** Hazard ratios (HRs) of death during different observation period after CRC surgery depending on the inflammatory properties of diet measured by the DII

	DII [continuous]				DII [>−2.27 vs. ≤−2.27]			
	1st year	3 years	5 years	Overall	1st year	3 years	5 years	Overall
Patients without distant metastases^a^								
Total number of observations	510	510	510	510	510	510	510	510
Number of failures [*n*, (%)]	23 (4.5 %)	79 (15.5 %)	125 (24.5 %)	150 (29.4 %)	23 (4.5 %)	79 (15.5 %)	125 (24.5 %)	150 (29.4 %)
Total time at risk (person-years)	480.96	1,287.37	1,926.53	2,906.10	480.96	1,287.37	1,926.53	2,906.10
HR	0.87	0.92	0.97	0.98	0.40	0.61	0.70	0.76
95 % CI	0.75–0.999	0.85–1.009	0.90–1.04	0.92–1.05	0.16–1.000	0.38–0.99	0.48–1.02	0.55–1.08
*p*	0.048	0.079	0.366	0.629	0.050	0.046	0.062	0.130
Patients with distant metastases								
Total number of observations	178	178	178	178	178	178	178	178
Number of failures [n, (%)]	68 (38.2 %)	151 (84.8 %)	159 (89.3 %)	159 (89.3 %)	68 (38.2 %)	151 (84.8 %)	159 (89.3 %)	159 (89.3 %)
Total time at risk (person-years)	141.60	236.89	258.96	274.21	141.60	236.89	258.96	274.21
HR	1.10	1.01	1.003	1.003	1.43	1.09	1.06	1.06
95 % CI	0.98–1.25	0.94–1.09	0.93–1.08	0.93–1.08	0.85–2.39	0.78–1.52	0.76–1.48	0.76–1.48
*p*	0.112	0.776	0.927	0.927	0.175	0.628	0.727	0.727

Multivariable Cox regression models—all estimations (models) were adjusted for age [years], smoking status [non-smoker, ex-smoker, current smoker], marital status [married, other], overweight or obesity [yes/no], calendar year when surgery was performed, surgery type [palliative/radical], cancer site [colon/rectum], chemotherapy after surgery [yes/no], radiotherapy after surgery [yes/no]

^a^One patient has been excluded from the analysis due to no information regarding anthropometric measurements (the presence of overweight or obesity)

## Discussion

Our study showed the beneficial effect of a long-term adult exposure to diet with lower pro-inflammatory properties (measured by the DII) among CRC patients treated surgically who had no distant metastases at the time of diagnosis and treatment. The association was observed in the first year after surgery and persisted until the third year. These results are consistent with our expectations. In our study, we were able to assess habitual diet before surgery, so the effect (if present) should be observed during relatively short period after surgical treatment. Additionally, the effect of diet as a determinant of death among CRC patients was supposed to be weak in the presence of distant metastases; therefore, the lack of an effect among patients in stage IV was not surprising.

**Fig. 2:**
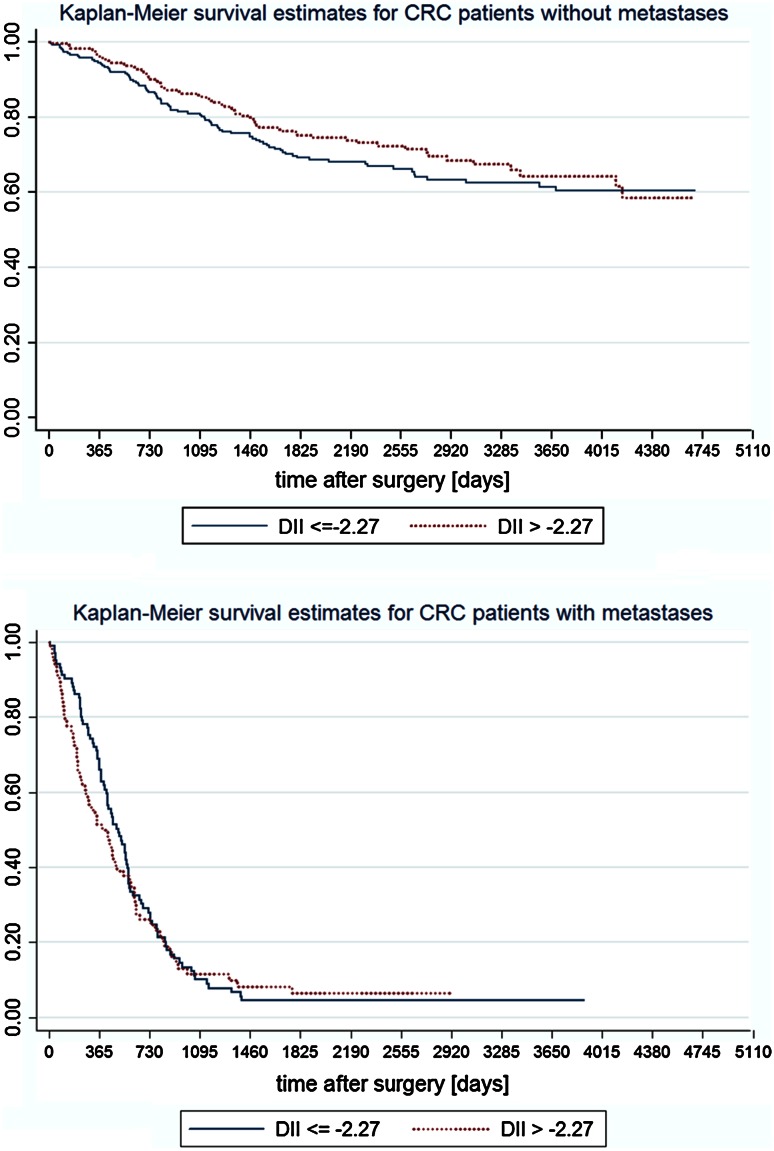
Kaplan-Meier survival curves in patients without and with distant metastases across categories of the DII

**Fig. 3:**
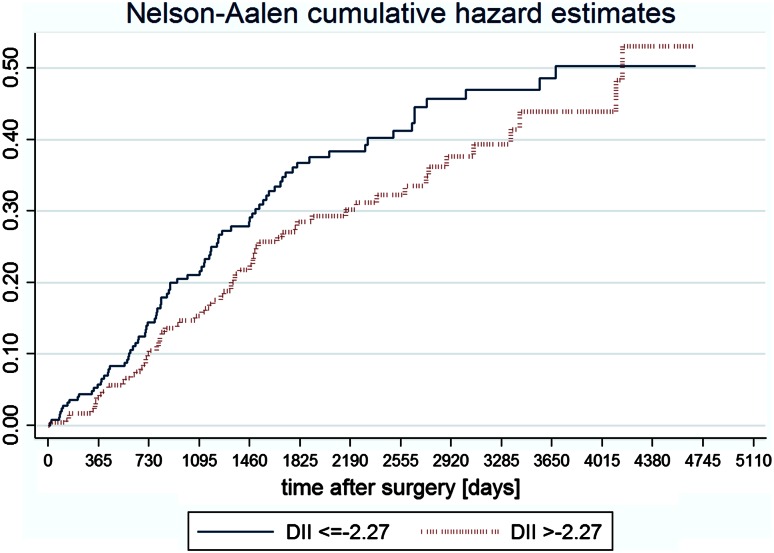
Nelson–Aalen cumulative hazard functions for patients without metastases across categories of the DII

The analysis of Kaplan–Meier survival curves (Fig. 2) has shown that the role of inflammatory properties of diet may have a long lasting effect (meaning longer than 3 years), but the reduction in sample size limited the possibility to prove it in a statistical way. The long lasting effect is also suggested by the Nelson-Aalen cumulative hazard functions (Fig. 3).

Although the presented concept is new, our results are strengthened by the cohort prospective design, an ability to include relatively large number of patients and a possibility to adjust for a large number of important covariates as age, smoking status, a type of surgery, cancer site, chemo and radiotherapy after surgery. Additionally, we considered the possibility of ‘the cohort effect’ related to the fact that patients treated in different calendar periods might undergo slightly different treatments due to some improvements in medical procedures. Therefore, our results were also controlled for a calendar year when patients were treated. We decided also to use a proxy measure (as marital status) of some social circumstances, which might determine the quality of care and the ability to fulfill treatment regimen after hospitalization. Finally, we used the presence of overweight and obesity as a covariate, because it was observed that an adipose tissue, particularly visceral fat, releases some pro-inflammatory cytokines, such as CRP, interleukin-6 (IL-6) and tumor necrosis factor-alpha (TNF-α) (Kwon and Pessin 2013), and in that way overweight and obesity might confound the relationship.

Our study has shown a decrease in the risk of death among patients with higher DII. These results require some elucidation. Firstly, we have noticed the effect only among patients without metastases, and the linear association was observed if patients were limited to those who had surgically removed cancer treated by chemotherapy. This is consistent with the concept that exposure to worse diet resulting in prolong low-grade inflammation may lead to a decrease in body defense and, as a consequence, may limit a possibility to maintain relatively good health during chemotherapy. Preventing and treating the toxicities of chemotherapy remains a major challenge for cancer treatment, and the general patient’s health condition related to the experiences before surgery may play a crucial role. The concept is also consistent with the results of some animal studies, showing that consumption of long-chain omega-3 fatty acids improved the therapeutic index of some cancer drugs (Hardman et al. 2002).

The role of inflammation among surgical patients has been investigated for several years. It was found that inflammatory status is among important markers of hospital morbidity and mortality. Consequently, immunomodulating diet was proposed as an intervention to improve clinical outcomes (Klek et al. 2011; Marano et al. 2013; Barker et al. 2013; Falewee et al. 2013; Hubner et al. 2012). There are also studies showing the role of inflammatory markers in survival. An elevated CRP was linked with poorer survival in CRC patients receiving adjuvant chemotherapy (Crozier et al. 2006). In the NHANES III cohort of 7,072 individuals, a clinically raised (≥1.00 mg/dl) baseline serum CRP was associated with approximately 2.5-times greater risk of CRC death (Swede et al. 2014). Leitch observed an increased risk of CRC death across categories of modified Glasgow Prognostic Scale among both 149 CRC patients without distant metastases and 84 CRC patients with unresectable colorectal liver metastases (HRs = 2.01, and 1.46, respectively) (Leitch et al. 2007). Read studied 23 CRC stage IV patients referred to chemotherapy, and he observed a significant positive correlation between survival time and serum concentration of IL-10 (*r* = 0.488, *p* = 0.03) and a negative correlation between survival time and a concentration of IL-6 (*r* = −0.63, *p* = 0.003) (Read et al. 2007). There is also some other evidence showing the role of inflammation in survival among CRC patients (Paik et al. 2014; Crozier et al. 2009; Nozoe et al. 2008).

The effect of inflammatory response to diet is definitely weaker than the response to the presence of a clinical disease and surgical treatment, which are determinants of inflammatory state before and after surgery. That is why we considered diet as an adult lifetime exposure responsible for the deterioration of a general condition among cancer patients rather than a point in time factor related to survival.

Dietary habits as a determinant of low-grade inflammation may play a role through its effect on levels of pro-inflammatory cytokines. Another possible explanation is that low-grade diet-related inflammation may be directly associated with the ARG1 pathway. It was observed that within hours of physical injury, large number of arginase-1 (ARG1)-expressing immature myeloid cells (IMCs) accumulate in the spleen and other lymphoid tissue (Makarenkova et al. 2006). These cells inhibit T lymphocyte growth and function, resulting in impairment of T cell proliferative response (Zhu et al. 2010). The preventive goal in this situation is to minimize the immunosuppression and to promote healing and tissue repair that is the rationale for immunomodulating diet suspected to improve surgical outcomes. Prolonged, low-grade inflammation may lead to the lack of reserves on a sub-clinical level, and after surgery and chemotherapy, it may be a risk factor for unfavorable outcomes among patients.

Our investigation is not free from limitations. Diet is constituted by much more than 23 dietary items we were able to consider. Nevertheless, we would like to mention that we included all relevant—those which are the main dietary components in our country. Next, we have considered habitual diet before surgery. As it is consistent with the concept of the life course approach (meaning the study of ‘person’s experiences over a life time that can have cumulative effects on their health’), after digestive tract surgery, patients usually change their diet. In our study, we had no data about dietary habits after surgery, which might also influence the survival. Moreover, pre- and post-surgery inflammatory response might also determine long-term consequences, but data about inflammatory markers were not available in our study either.

In summary, our investigation of the inflammatory properties of habitual diet using the DII as a predictor of survival among CRC patients is novel to our knowledge, and it provides a new insight into the role of diet. The results of the current research have shown the usefulness of the DII developed by Cavicchia as a potential predictor of survival among patients without distant metastases treated surgically for CRC. Moreover, by more complex assessment of the role of diet considering its inflammatory properties, it provides the evidence for the role of dietary habits, and if confirmed by other investigations, that may explain some heterogeneity in patients’ survival outcomes.

## Appendix

See Table 3.

**Table 3:** Adjusted scores for dietary constituents used for the analysis (scores according to Cavicchia et al. 2009)

Constituent	Adjusted score	Measure
Energy	−0.23	kcal/day
Tea	0.552	g/day
Caffeine	0.035	g/day
Wine	0.48	g/day
Beer	0.2	g/day
Alcohol	0.534	g/day
Carbohydrate	−0.346	g/day
Fiber	0.52	g/day
Fat	−0.323	g/day
n-3 fatty acids	0.384	g/day × 10
n-6 fatty acids	−0.016	g/day × 10
MUFA	−0.05	g/day
Saturated fatty acids	−0.25	g/day
Protein	0.05	g/day
Cholesterol	−0.21	mg/day
Vitamin A	0.58	µg/day ÷ 100
Thiamin	0.05	mg/day
Riboflavin	0.16	mg/day
Niacin	0.26	mg/day
Vitamin C	0.367	mg/day
Vitamin E	0.401	mg/day
Β-Carotene	0.725	µg/day ÷ 100
Iron	0.029	mg/day

*MUFA* monounsaturated fatty acids

## Abbreviations

ARG: Arginase
CRC: Colorectal cancer
CRP: C-reactive protein
DII: The Dietary Inflammatory Index
IL: Interleukin
IQR: Inter-quartile range
MUFA: Monounsaturated fatty acids
TNF: Tumor necrosis factor
